# Case of Psora Treated with Tobacco

**Published:** 1812-01

**Authors:** 


*To the Editors of the Medical and Physical Journal.*

GENTLEMEN,

KNOWING your publication to be the avowed repository for every subject, whether curious or useful, practical or speculative, that relates to medical literature, I have sent you the following, with a hope that you will indulge it with a place in your next number.

A young man of my acquaintance, being unfortunately infected with Psora, applied to me for relief, wishing me to compound for him some unguent which contained no sulphur, by reason of the inconvenience resulting from its its disagreeable smell. Having in vain endeavoured to conquer his prejudices by representing to him how precarious and unsafe such a mode of treatment was, generally considered, I resolved to make use of an infusion of tobacco, a remedy strongly recommended by Dr. Thomas, of Salisbury, in the third edition of his excellent work, intitled "The Modern Practice of Physic." Accordingly, he was fomented from head to foot with an infusion of this herb, made according to the directions of the last London Pharmacopœia. At the expiration of about twenty minutes, he complained of nausea; but, not anticipating any ill consequences from the operation alluded to, I judged this to be merely the effect of a redundant secretion of bile, a disease to which the patient was exceedingly prone. However, I was soon convinced of my mistake. In less than five minutes after, in addition to nausea, he complained of head-ache, vertigo, stupor, and universal debility. I now saw enough to convince me, that the patient's indisposition was caused by the tobacco, and, accordingly, the fomentation was immediately discontinued. The symptoms however increased, and obliged me to think of administering some appropriate medicine for his relief.

Considering the stomach as the organ primarily affected, and vomiting being indicated by the perpetual nausea which prevailed, I resolved to give a gentle emetic of ipecacuanha; but, before this could be procured, the patient, on a sudden, began to vomit copiously, and continued to do so at intervals for several hours. After the vomiting ceased, he felt much recovered, and, on the following morning all the unpleasant symptoms left him. He continued, however, in a state of extreme debility; which, perhaps, might have been accounted for by the violent and long-continued exertion of the stomach, and the impaired appetite which necessarily ensued. To restore the loss of tone in this organ, and invigorate the system in general, I gave the vegetable tonics, such as bark, calumba, &c.; now and then a gentle saline purgative, for the purpose of washing off any offending matter which might have accumulated in the intestines. With this treatment the patient returned to convalescence in about five days.

It may perhaps be thought easy to account for this singular circumstance, by commencing with the absorption of the tobacco infusion by the lymphatics of the cutis, and tracing its subsequent progress through the vascular system. Not choosing however to risk the accusation of either presumption or mistake, I shall leave that subject to such of your your intelligent correspondents who are more minutely versed in the laws of the animal œconomy.

And remain, Gentlemen,

Your most obedient Servant,

STUDIOSUS MEDICINÆ.

*Tilshead, near Devizes, Wilts. Nov.* 6, 1811.

